# Ecological niche modelling of Hemipteran insects in Cameroon; the paradox of a vector-borne transmission for *Mycobacterium ulcerans*, the causative agent of Buruli ulcer

**DOI:** 10.1186/1476-072X-13-44

**Published:** 2014-10-25

**Authors:** Kevin Carolan, Solange Meyin À Ebong, Andres Garchitorena, Jordi Landier, Daniel Sanhueza, Gaëtan Texier, Laurent Marsollier, Philipe Le Gall, Jean-François Guégan, Danny Lo Seen

**Affiliations:** Unité mixte de recherche (UMR) Maladies Infectieuses et Vecteurs : Écologie, Génétique, Evolution, et Contrôle (MIVEGEC) IRD-CNRS-Universities of Montpellier I and II, Centre IRD de Montpellier, BP 64501, 34394 Montpellier Cedex 5, France; UMR Territoires, Environnement, Télédétection et Information Spatiale (TETIS) CIRAD, 34093 Montpellier, France; Laboratoire de Mycobactériologie, Centre Pasteur du Cameroun, Réseau International des Instituts Pasteur, Yaoundé, Cameroun; Ecole des Hautes Etudes en Santé Publique, Rennes, France; Unité d’Epidemiologie de Maladies Emergentes, Institut Pasteur, 25 Rue du Docteur Roux, 75015 Paris, France; Service d’épidémiologie et de santé publique, Centre Pasteur du Cameroun, Réseau International des Instituts Pasteur, Yaoundé, Cameroun; UMR 912 - SESSTIM - INSERM/IRD/Aix-Marseille Université Faculté de Médecine, 27, Bd Jean Moulin, 13385 Marseille, France; ATOMycA, Inserm Avenir Team, CRCNA, Inserm U892, 6299 CNRS and LUNAM, Angers, France; IRD, Institut de Recherche pour le Développement, UR 072, Laboratoire Evolution, Génomes et Spéciation, UPR 9034, Centre National de la Recherche Scientifique (CNRS), 91198 Gif sur Yvette Cedex, France et Université Paris-Sud 11, 91405 Orsay Cedex, France

**Keywords:** Ecological niche modelling, Naucoridae, Belostomatidae, Spatial distribution, Habitat suitability, Buruli ulcer, *Mycobacterium ulcerans*, Vector-borne transmission, Environmentally-acquired disease

## Abstract

**Background:**

The mode of transmission of the emerging neglected disease Buruli ulcer is unknown. Several potential transmission pathways have been proposed, such as amoebae, or transmission through food webs. Several lines of evidence have suggested that biting aquatic insects, Naucoridae and Belostomatidae, may act as vectors, however this proposal remains controversial.

**Materials and methods:**

Herein, based on sampling in Cameroon, we construct an ecological niche model of these insects to describe their spatial distribution. We predict their distribution across West Africa, describe important environmental drivers of their abundance, and examine the correlation between their abundance and Buruli ulcer prevalence in the context of the Bradford-Hill guidelines.

**Results:**

We find a significant positive correlation between the abundance of the insects and the prevalence of Buruli ulcer. This correlation changes in space and time, it is significant in one Camerounese study region in (Akonolinga) and not other (Bankim). We discuss notable environmental differences between these regions.

**Conclusion:**

We interpret the presence of, and change in, this correlation as evidence (though not proof) that these insects may be locally important in the environmental persistence, or transmission, of *Mycobacterium. ulcerans*. This is consistent with the idea of *M. ulcerans* as a pathogen transmitted by multiple modes of infection, the importance of any one pathway changing from region to region, depending on the local environmental conditions.

## Background

The Buruli ulcer is an emerging neglected tropical disease affecting more than 5,000 people per year in West and Central Africa, French Guiana, Latin America and Australia [1]. The disease burden is highest in Africa, where it predominantly affects children under the age of 15, and due to damage to the skin, muscle and bone, can result in severe scarring and crippling deformities if left untreated. The disease is caused by the environmental pathogen *Mycobacterium ulcerans*.

The mode of transmission of *M. ulcerans*, the method by which it infects humans, is unknown. Many routes of transmission have been proposed, such as transmission by aerosol [2], vector transmission by amoebae [3] or through aquatic networks [4]. During a study of the association between *M. ulcerans* and aquatic plants in Ghana and Benin aquatic insects were accidentally collected during the sampling procedure, and unexpectedly found to test positive for *M. ulcerans* [1]. The authors proposed that, given that these insects occasionally bite humans, they may be implicated in transmission of *M. ulcerans*. Aquatic insects have been further implicated after a series of laboratory experiments demonstrated the competency of Naucoridae to act as vectors. Naucoridae are able to acquire *M. ulcerans* from their diet, and then transmit the pathogen to mice resulting in Buruli-like symptoms [5–8]. Buruli ulcer is commonly associated with lowland, stagnant water [9] and human behaviours associated with water bodies appear to be risk factors for Buruli ulcer infection, which would lend support to the idea of infection occurring in an aquatic context.

However, the role of these insects has been disputed for several reasons. In a two year study of Buruli ulcer endemic and non-endemic sites in Ghana [10], found no evidence for a role in transmission. The population of these insects, and the prevalence of *M. ulcerans* infection in them, was not significantly different between Buruli ulcer endemic and Buruli ulcer non-endemic sites [10]. The authors argued that, if these insects are vectors, we would expect them to have a higher abundance in Buruli ulcer endemic sites, that rates of *M. ulcerans* infection of the insects should be higher in Buruli ulcer endemic sites, and that the rate of infection of these insects should be higher than other species of invertebrates.

These expectations are based on the Bradford-Hill guidelines for associating insect vectors with human vector-borne disease [11, 12]. These guidelines provide a general framework to explore the association between vectors and disease, based on the consistency, specificity, plausibility and coherence of the proposed mode of transmission.

Consistency refers to the expectation that the rate of infection of the proposed vectors, which should be consistently strongly positively associated, in time and space, with prevalence of human cases. This also implies human cases should not occur in absence of the proposed vector. The proposed vector should have a demonstrated capacity to physically transmit the pathogen, which has been demonstrated for *M. ulcerans* in the lab [5]. The interaction between the proposed vector and human infection must be specific and alternative explanations of human infection should be ruled out (though see [4] for alternative explanations). That is, human infection must be demonstrated to not have been the result of other potential modes of transmission. We note that this criterion must be applied with care in cases of multi-host transmission.

Additionally, the proposed vector must plausibly be able to be a vector of the pathogen. This criterion is often controversial as it is highly dependent upon the experience of the researcher, and their opinion about what is, and is not, plausible as opposed to merely possible [13]. Most authors in Buruli ulcer research would agree with some basic facts; the waterbugs are infected in the environment [14], they bite humans occasionally, and are able to transmit the bacteria to mice in the lab [5]. However, waterbugs are not known to be vectors of other pathogens, and related Mycobacteria (*Mycobacterium tuberculosis, M. leprae, M. marnium*) are not known to be vector-borne diseases [10, 12]. The plausibility of this proposed route of transmission is still debated. The final criterion, coherence, is based on what we already know about the pathogen, the vector and the host. Does the proposed method of transmission fit well with our current understanding of its biology? As our understanding of the biology of *M. ulcerans* improves, this criterion will be answered.

Given this framework, how likely is it that Naucoridae and/or Belostomatidae are vectors of the Buruli ulcer disease? Herein, we explore the correlation in time and space between the proposed vectors, Naucoridae and Belostomatidae, and the Buruli ulcer prevalence. We discuss the other Bradford-Hill criteria, but do not focus on them specifically, as it was not directly within the scope of this work. Based on sampling in Cameroon, we characterise the set of suitable habitats within which species of the Families Belostomatidae and Naucoridae can maintain a population (their ecological niche) and describe the spatial distribution of these suitable habitats across West Africa. We then explore any correlations between habitat suitability and Buruli ulcer prevalence at multiple spatial scales.

## Materials and methods

### Distribution of Belostomatidae and Naucoridae aquatic insect families

**Figure 1:**
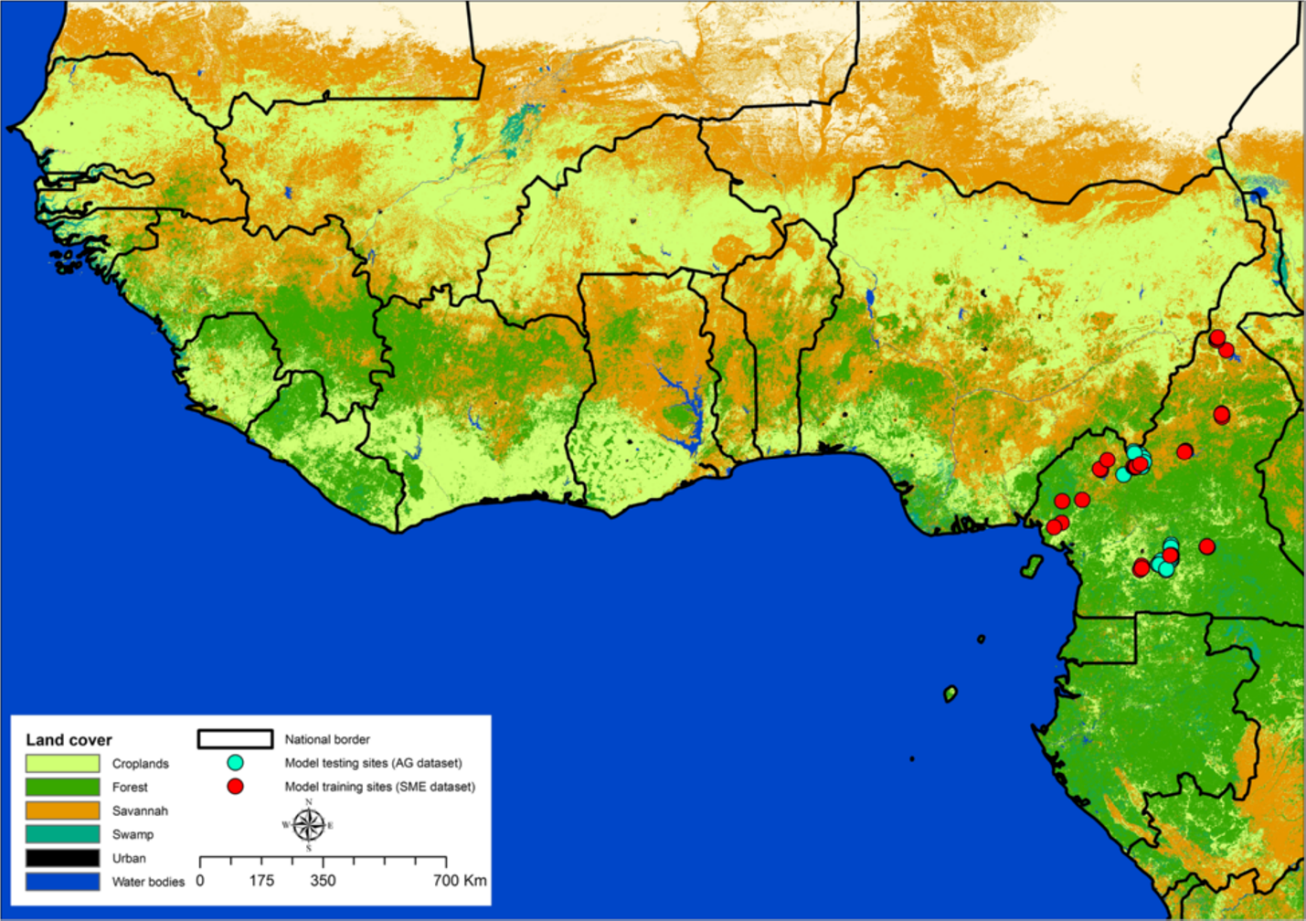
**Study sites Cameroon against local land cover.** Data from the SME dataset is in red, and AG data set is in blue. The sample sites span the extent of Cameroon, sampling from every major land cover category.

Data were collected as described in [15] hereafter referred to as the SME dataset. In brief, 36 sample sites in Cameroon were visited monthly from September 2012 to February 2013 (Figure 1), a period including both wet and dry seasons. Dip net sampling was conducted at all sites. Due to limitations of current taxonomic keys, the aquatic insects of interest were only identifiable to the phylogenetic division of Family. A second dataset was used in model validation, using data collected separately by A. Garchitorena (Figure 1). This dataset was collected as described in [16], and is hereafter referred to as the AG dataset. Niche models of Belostomatidae and Naucoridae are constructed using the SME dataset, and then tested on their ability to reproduce the independent AG dataset.

### Environmental parameters used in ecological niche modelling

Five ecological parameters were used to describe the distribution of suitable habitats: rainfall, flow accumulation, wetness index, land cover at the sample site, and land cover within the flight range of adult insects. These variables were selected on the basis of their likelihood to influence the distribution and condition of water, and are summarised in Table 1. Rainfall was highly seasonal, so we divide models by the season of collection. Models constructed using species distribution data from the dry season used the precipitation in the driest season; models constructed using species distribution data from the wet season used the precipitation in the wettest season. These two variables were taken from the Worldclim database, as BIO13 Precipitation of Wettest Month and BIO14 Precipitation of Driest Month [17]. Flow accumulation was derived using elevation data [18]. Flow accumulation is the surface area contributing water to a particular point, and indicates the potential amount of water available, which is then determined by rainfall. Using the SRTM elevation, flow accumulation was derived using the Fill, Flow Direction and Flow Accumulation tools in ArcMap 10.1 [19]. Wetness index has previously been shown to be associated with the Buruli ulcer [9]. In ecological terms it indicates the topological potential for water to accumulate, and was derived according to Equation 1,

**Table 1:** **Environmental variables used in ecological niche modelling**

Variable	Units	Original resolution	Source
Wetness index	m^2^	15 arc-sec (approx 450 m^2^)	SRTM
Flow accumulation	m^2^	15 arc-sec (approx 450 m^2^)	SRTM
Precipitation in wettest season	millimeter	30 arc-sec (approx 1 km^2^)	Bioclim 13
Precipitation in driest season	millimeter	30 arc-sec (approx 1 km^2^)	Bioclim 14
GLC-AP	Unitless	300 m^2^	GLC 2009
GLC-5 K	Unitless	300 m^2^	GLC 2009

All data were resampled to a spatial resolution of 0.004 decimal degrees (~300 m^2^) for use in Maxent. Resampling used *resample()* in the library ‘raster’ of the software R.

1

where *FA* was the flow accumulation, 500 was the cell size in meters, and *S* was the surface slope in degrees. Large flow accumulation values and flat slopes resulted in high wetness index values, and indicate areas where water is likely to stagnate. In areas where the slope is zero wetness index had no value. Land cover was derived from the Global Land Cover Map 2009 [20]. GLC-At Point (hereafter GLC-AP) is the land cover at the sample site. In a 5 km radius around the site the most common (modal) land cover category was described. For example, a sample site may be in savannah, but surrounded by forest. 5 km was selected as the approximate flight radius of the insects [21–23]. This is termed GLC-5 K.

### Data preparation, niche modelling, and prediction of spatial distribution of suitable habitat

Insect distribution data from the 36 sample sites (Figure 1) were explored to identify normality, homogeneity of variance and correlation in the five environmental parameters used. Two sites were excluded from analysis as apparent outliers in flow accumulation, otherwise the data were normally distributed and homogenous. Correlation was observed between flow accumulation and wetness index and between precipitation in the driest and in the wettest seasons, however this was not significant (*p* > 0.05) using a Spearman’s correlation test. All data were resampled to a spatial resolution of 0.004 decimal degrees (~300 m^2^) for modelling using *resample()* in the library ‘raster’ of the software R [24].

Across the scale of West Africa we assume that absence data is not reliable, as it is more likely to indicate failure of detection rather than evidence of absence. For this reason, we chose to conduct presence-only modelling, and the specific method selected was Maximum Entropy [25]. We used the software Maxent (Maxent 3.3.3 k, [25]) to construct these models. Maxent has been used several times in the past to model the ecological niche of disease vectors [26]. Maximum entropy modelling minimises the divergence between the distribution of the environmental parameters and the species distribution, assuming the species is distributed in the most ecologically efficient manner possible. Maxent describes the environmental parameters across the study region, and is sensitive to the size of the study region. Herein, we generated background points in every raster cell within the extent of our study (that is, every environment was described). The models were replicated 100 times and averaged.

**Figure 2:**
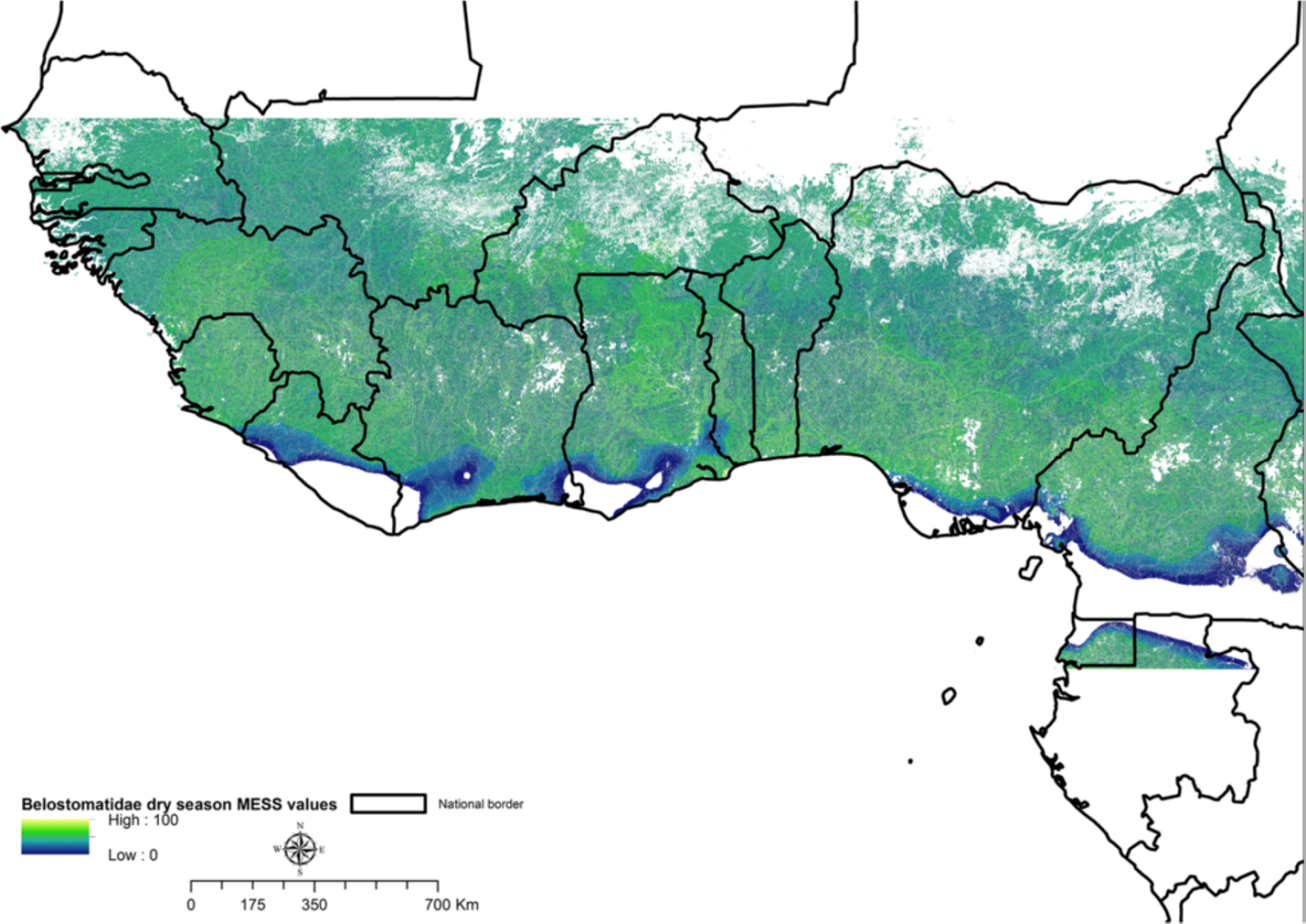
**Delineation of the study area.** We exclude areas of ecological extrapolation. Across West Africa the red-green coloured region is ecologically interpolative within our sites, according to a multivariate environmental similarity surface (MESS). Regions where we extrapolate beyond the ecology of our study sites are identified using MESS values less than 0. Colour values are MESS values, indicating similarity to sample sites; a value of 100 is identical to the median of the sample sites. The spatial projection of this interpolative region is shown against a map of country borders of West Africa, for Belostomatidae adults in the dry season. This projection changes for each family, developmental stage and season, and though it is spatially discontinuous it is ecologically homogenous.

In order to select an appropriate extent to the area considered for modelling we chose to confine the spatial extent of our study to the region ecologically interpolated by the sample sites (Figure 2). The sample sites of the SME dataset describe a particular range of environmental conditions for the five environmental parameters (Table 1). We excluded conditions that were not studied by the SME regime (much larger or lower values of rainfall, land cover categories not sampled, flow accumulation not observed at the study sites) to avoid extrapolating beyond the range of ecological parameter values studied. To select this interpolative region we constructed a Multivariate Environmental Similarity Surface (MESS map [27]), excluding areas with negative MESS values from our study region. A MESS map was constructed for each model; models with more samples can be interpolated into larger ecological regions. MESS maps were constructed with the function *mess()* available in the library *dismo* in R.

### Evaluating model performance

We evaluated the performance of these models according to their ability to correctly predict the distribution of the insects (with a separate dataset, the AG dataset) as efficiently as possible (avoiding overfitting). We used two methods to evaluate the models. First, the Akaike information criterion, corrected for small sample size (AICc), was used. AICc was generated as in [28], and penalises complex models to avoid over-fitting the data, low AICc scores indicate the model is not over-fitting. Second, we tested the models ability to predict the AG dataset. The Area Under Curve (AUC) is often used to evaluate Maxent models, and has been criticized previously [29]. We used a modified version of the AUC for model evaluation, termed here AG AUC. The model predictions were compared to the known values as collected by AG. AG AUC values range from 0 to 1, values close to 1 indicate good performance, 0.5 is no better than random. Use of the AG datas*et* allowed us to use true absence data for model validation, avoiding the problem of pseudo-absence data in Maxent.

The purpose of these two different metrics is to consider different aspects of performance, neither were without limitations. Use of the AG dataset allowed a degree of validation across methodologies, indicating the extent to which our results were dependent on a particular sampling regime. AICc is traditionally used to indicate over-fitting in models, however it is sensitive to the size of the ecological niche of the species.

### Identifying the relationship between habitat suitability and Buruli ulcer prevalence

**Figure 3:**
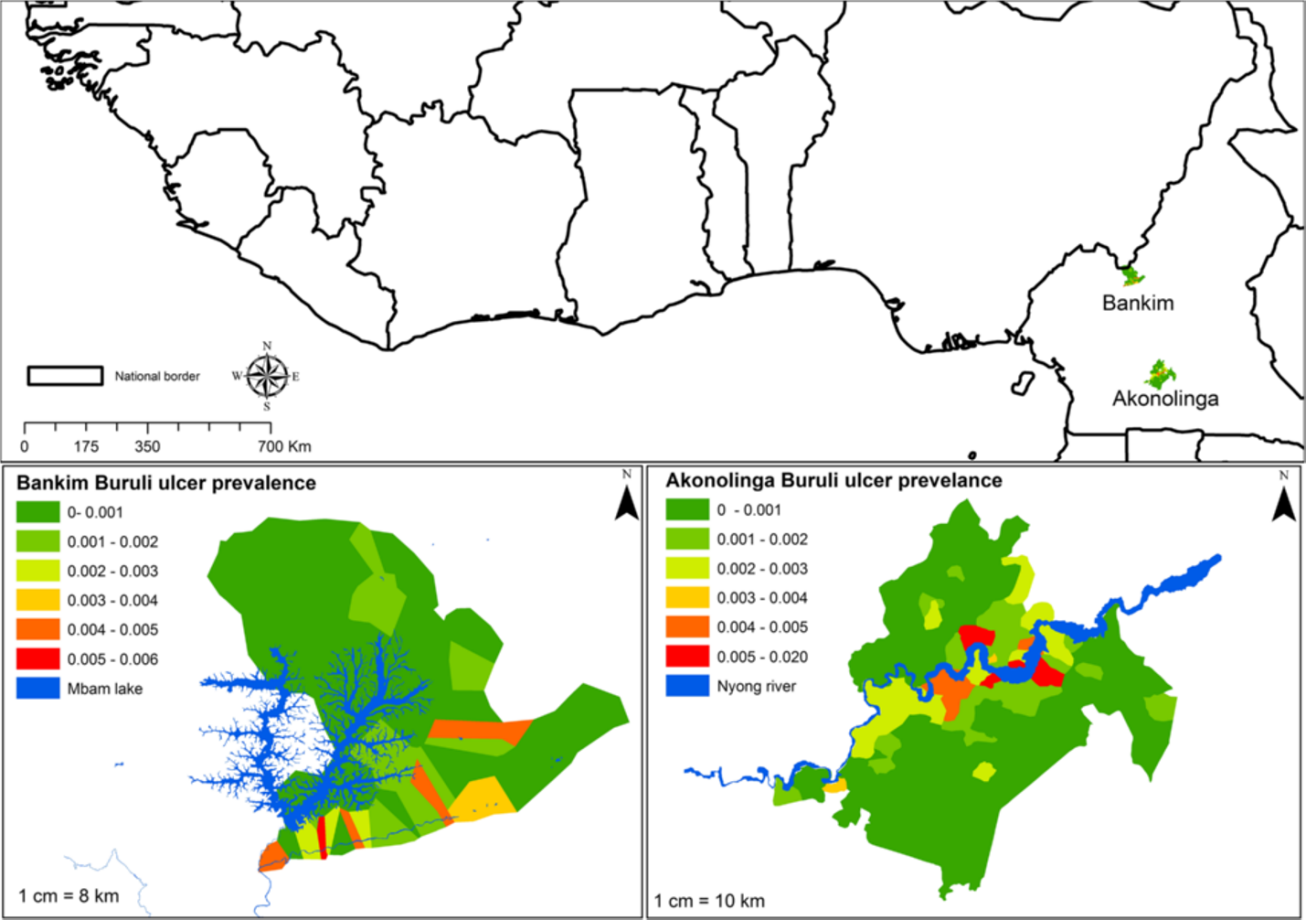
**Spatial distribution of Buruli ulcer prevalence in Akonolianga and Bankim, two endemic regions within Cameroon.**

Buruli ulcer prevalence data was collected for two endemic regions in Cameroon, Akonolinga [30] and Bankim [31], as shown in Figure 3. Around the centre of each village a buffer was created, and average habitat suitability in this buffer was correlated to the village Buruli ulcer prevalence using Spearman’s rank correlation coefficient. Seven buffers were used to explore the effect of buffer size and shape. Around the centre of the village circular buffers of 1, 2, 3, 4, 5 and 10 km were selected, and average habitat suitability recorded. We also used a buffer defined by the borders of the village (Figure 3). This buffer changes in size for each village, and represents the approximate extent of the village area. 5 km is approximately the flight radius of the insects, and the distance easily walkable by local people on an average day in Akonolinga [32]. To deal with this multiple testing, Bonferonni’s correction of the *p*-values was used.

## Results

### Distribution of suitable habitat, and its relationship to Buruli ulcer

**Figure 4:**
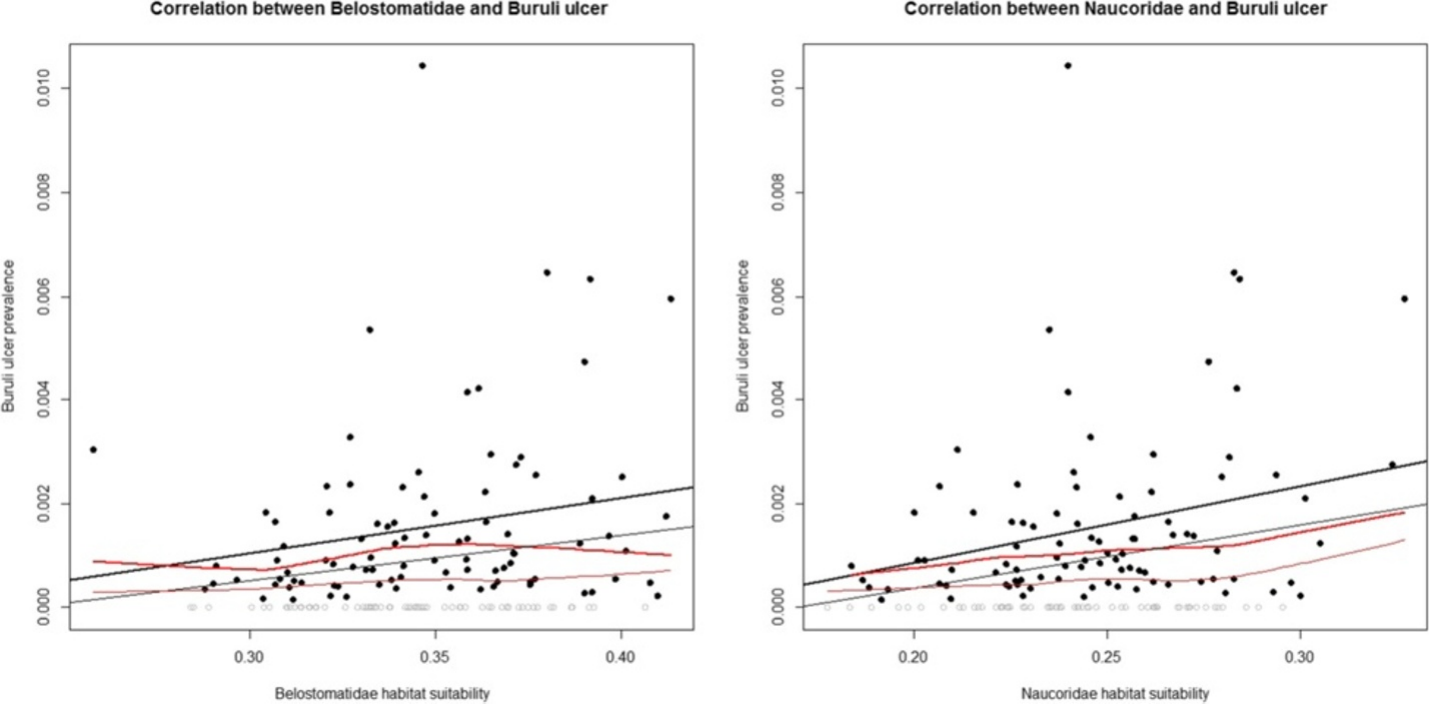
**Correlation between the prevalence of Buruli ulcer and habitat suitability of Belostomatidae (left) and Naucoridae (right) in the wet season in Akonolinga.** Colour indicates use of a linear model (Black) or locally weighted scatterplot smoothing (Red), different ways of viewing the correlation. Buruli ulcer was absent from certain villages (grey dots) where habitat suitability for the insects is high. Because this can skew any correlation between habitat suitability and prevalence, we explored the effect of including (thin lines) or excluding (thick lines) these villages. We note that in either case Spearman’s rank correlation coefficient was significant. For Belostomatidae with Buruli ulcer absent villages *p* = 0.08, without BU absent villages *p* = 0.03. For Naucoridae with BU absent villages *p* = 0.04, without BU absent villages *p* = 0.02.

In Akonolinga there was a significantly positive correlation between Buruli ulcer prevalence and average habitat suitability, for both Naucoridae and Belostomatidae, in the wet season (Table 2, Figure 4). This relationship was significant at multiple buffer distances. In contrast, in Bankim there was no significant correlation between Buruli ulcer prevalence and Belostomatidae or Naucoridae average habitat suitability, in either wet or dry seasons or at any buffer distance (Table 3).

**Table 2:** **Spearman’s rank correlation coefficients for correlation between Buruli ulcer prevalence and habitat suitability in Akonolinga, for both seasons and species**

Species	Season	Buffer	Bonferroni ***p***value
Belostomatidae	Dry	10	-
		5	-
		4	-
		3	-
		2	-
		1	-
		Village	-
	Wet	10	0.000
		5	0.014
		4	0.023
		3	-
		2	-
		1	-
		Village	-
Naucoridae	Dry	10	-
		5	-
		4	-
		3	-
		2	-
		1	-
		Village	-
	Wet	10	0.001
		5	0.013
		4	0.014
		3	-
		2	-
		1	-
		Village	-

The column labelled Buffer is the distance, in km, around the village centre that the habitat suitability is considered, the buffer labelled village uses village boarders as a buffer (Figure 3). Bonferonni’s *p* value is the significance of the correlation between the insect and the disease, for clarity only significant values (<0.05) are presented, non-significant values are marked “-”. Significant positive correlations were observed between both Belostomatidae and Naucoridae in the wet season and Buruli ulcer prevalence, but not the dry season.

**Table 3:** **Spearman rank correlation coefficients for correlation between Buruli ulcer prevalence and habitat suitability in Bankim, for both seasons and species**

Species	Season	Buffer	Bonferroni ***p***value
Belostomatidae	Dry	10	-
		5	-
		4	-
		3	-
		2	-
		1	-
		Village	-
	Wet	10	-
		5	-
		4	-
		3	-
		2	-
		1	-
		Village	-
Naucoridae	Dry	10	-
		5	-
		4	-
		3	-
		2	-
		1	-
		Village	-
	Wet	10	-
		5	-
		4	-
		3	-
		2	-
		1	-
		Village	-

The column labelled Buffer is the distance, in km, around the village centre that the habitat suitability is considered, the buffer labelled village uses village boarders as a buffer (Figure 3). Bonferonni’s *p* value is the significance of the correlation between the insect and the disease, for clarity only significant values (<0.05) are presented, non-significant values are marked “-”. No significant correlations were observed in Bankim.

### Ecologically important variables in the distribution of the aquatic insect families

**Figure 5:**
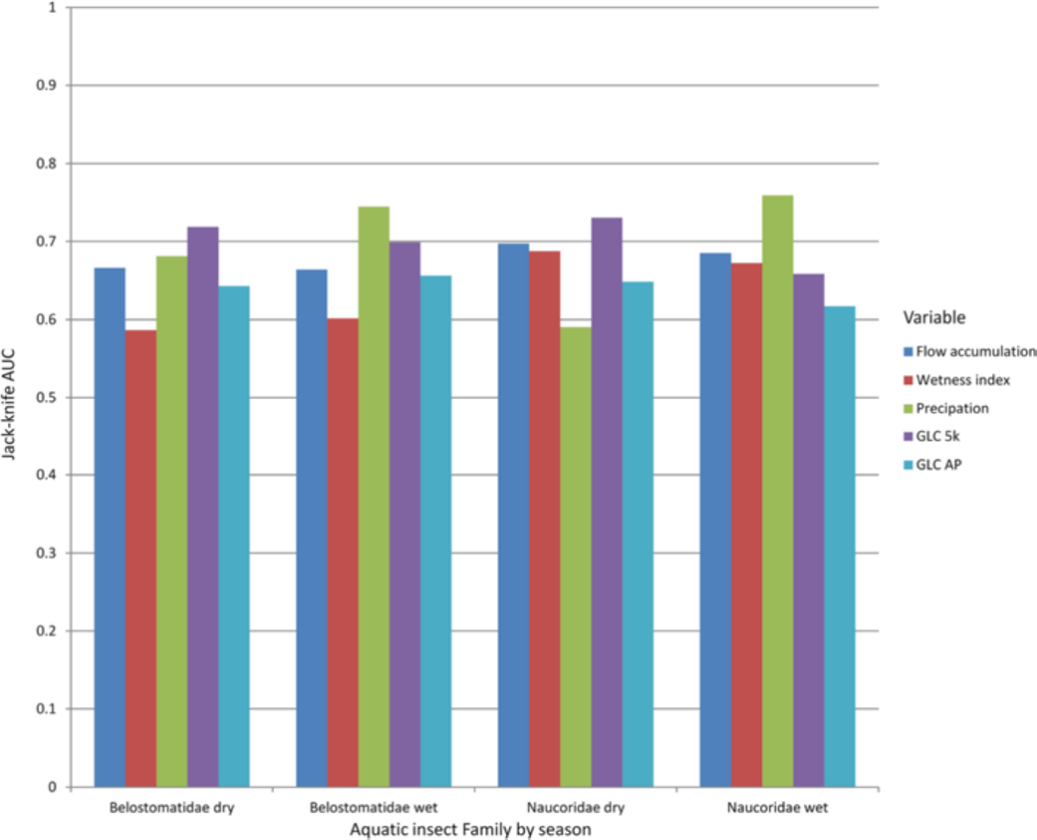
**Importance of each variable according to Jack-knife AUC for wet and dry seasons.** A high value indicates the variable is important; however this is sensitive to correlation within the variables. For both insects the most important variable in the dry season was the land cover in the flight radius (GLC 5 km), in the wet season precipitation was the most important variable.

**Figure 6:**
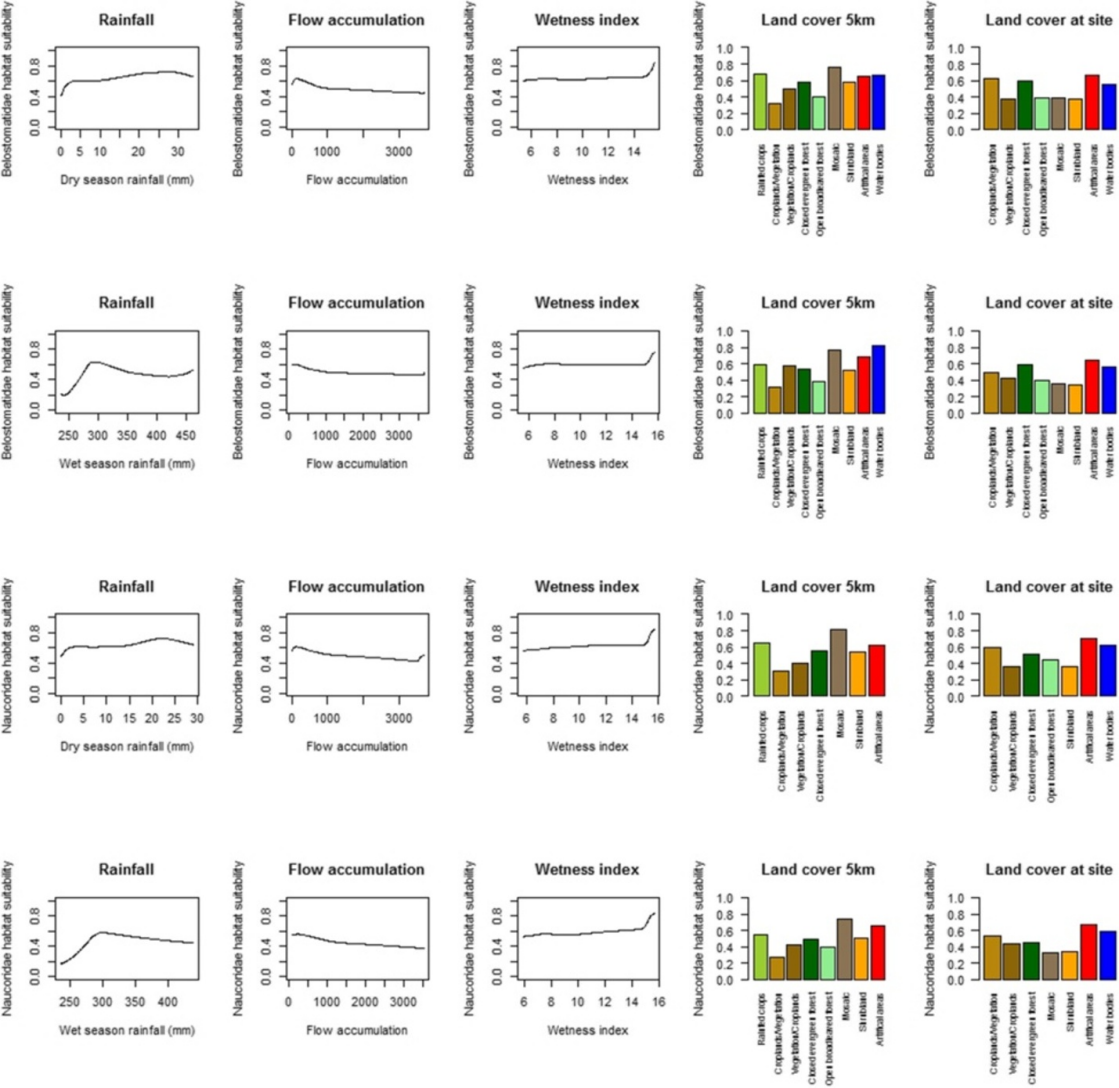
**Habitat suitability for Belostomatidae (1** ^**st**^ **and 2** ^**nd**^ **rows) and Naucoridae (3** ^**rd**^ **and 4** ^**th**^ **rows) in the wet and dry seasons.** Both insects have a negative relationship with flow accumulation, and a positive relationship with wetness index; an unexpected relationship given that increasing flow accumulation normally means increasing wetness index.

Variable importance was evaluated using Jackknife variable removal. Jacknife removes a variable and evaluates the effect of variable removal on the model. In the dry season Belostomatidae and Naucoridae responded in broadly similar fashions; the variable whose removal had the largest effect was GLC 5 km (Figure 5). The land cover categories most suitable for both Belostomatidae and Naucoridae are water bodies, artificial areas, rain fed croplands and forest/grassland mosaics (Figure 6). If one of these categories is the dominant category in 5 km radius, in the dry season, the likelihood of encountering the insect is higher. Unsuitable categories were forest and vegetation/cropland mosaic.In the wet season precipitation is more important than land cover. Precipitation suitability peaks at approximately 300 millimeters per month, and diminishes above or below this (Figure 6). For the dry season there is a simple increase in habitat suitability with increasing precipitation.

Flow accumulation had a negative association with habitat suitability, and wetness index had a positive association, regardless of season, for both Belostomatidae and Naucoridae.

### Model performance

AICc for Naucoridae adults (dry season) was 14.6, and 14.2 in the wet season, as in Table 2. For Belostomatidae adults (dry season) the AICc was 12.5, and 12.2 in the wet season. Scores of overfitting are relative; these scores indicate the Belostomatidae model was less prone to overfitting than the Naucoridae model.

The AG data set was also used in model validation. In the dry season Naucoridae adults had an AG AUC of 0.83, and 0.80 in the wet season. Belostomatidae adults had an AG AUC of 0.80 in the dry season, and 0.86 in the wet season. These scores indicate that the models are able to describe the distribution of the insects with good accuracy; the model based on SME dataset is able to accurately replicate the independently collected AG dataset.

## Discussion

We explored the correlation between the distribution of Belostomatidae and Naucoridae and the prevalence of Buruli ulcer. We have found a positive gradient between habitat suitability of Naucoridae and Belostomatidae and Buruli ulcer prevalence. Correlation does not imply causation; this result is not proof that the insects are vectors. However, understanding the reasons for the temporal and spatial changes in this correlation will enable a better understanding of the reasons for changes in Buruli ulcer prevalence.

There are significant temporal changes in this correlation between habitat suitability of the insects and Buruli ulcer prevalence; in Akonolinga the Buruli ulcer prevalence is correlated to Naucoridae and Belostomatidae distribution in the wet season, but not in the dry season. Buruli ulcer is known to have complex temporal changes in prevalence [30, 33, 34], as is *M. ulcerans* [14, 35]. It is therefore unsurprising that, if these insects are implicated in maintaining *M. ulcerans* in the environment, or involved in transmission to humans in some way (either as host vectors, or host carriers), the correlation between Buruli ulcer prevalence and their abundance would change in time.

We also observe spatial changes in the correlation; water bug habitat suitability is not correlated to Buruli ulcer prevalence in Bankim, 457 km North of Akonolinga. Speculatively, perhaps other routes of transmission may be more important in this region, for example contact with infected plant biofilms, as suggested in Ghana [36].

How do we interpret this result in terms of the Bradford-Hill guidelines? Herein we have focused on the correlation between these insects and the prevalence of the disease in both space and time. While there is a significant positive correlation for the predicted abundance of the aquatic insects and the prevalence of the Buruli ulcer, this correlation is not consistent from region to region. Previous research has proposed that *M. ulcerans* is transmitted within a multi-host transmission network [4]. In such a situation of multiple hosts the relative importance of any given mode of transmission may be expected to vary in time or space, and our results are consistent with, though not proof of, this hypothesis. The lack of a clear signal between water bugs and Buruli ulcer in Bankim would suggest that may not be key vectors or host carriers in that region. Recent studies have found notable changes in community composition relevant to *M. ulcerans* distribution, in the Greater Accra, Ashanti and Volta regions of Ghana, for both plant [35] and aquatic insects [37]. This would support the importance of changing biotic communities as a key factor in changing Buruli ulcer prevalence, a priority for future work. We have found that the prevalence of Buruli ulcer is correlated to Belostomatidae and Naucoridae abundance in Akonolinga but not Bankim, we do not know if the plant community composition for these regions, and wider aquatic insect community, also correlates to disease prevalence changes on a similar scale.

The ecological niches of both Naucorid and Belostomatid water bugs in West Africa are predominantly determined by the distribution of suitable landcover in a 5 km radius, preferring water bodies, artificial areas and rain fed croplands. The specific land cover at the point of the site (GLC-AP) was less informative. The observation that the most suitable regional land cover class is water bodies is not surprising, but the high suitability of urban areas is curious. Ecologically this could have a variety of causes; there may be changes in the chemical composition of water in these habitats, a reduction in predation pressure, or a greater abundance of food. The specific reasons will require further research.

Our study has been limited in certain points; the low taxonomic resolution of the insects is a current limitation in this study. Secondly, an important limitation is that the distribution of *M. ulcerans* in these insects in these areas is unknown. The distribution of Naucoridae and Belostomatidae infected by *M. ulcerans* may differ from the distribution of Naucoridae and Belostomatidae generally. However, the insects are known to host the bacillus on their carapace, in their body [10, 14, 16]) and in their salivary glands [14] in the wild, and the distribution of the insect necessarily sets a limit to the distribution of infected insects. A related limitation is the unknown incubation time of *M. ulcerans*; the time from infection to presentation at the hospital, remains unknown. Finally, we have only addressed a single criterion of the Bradford-Hill guidelines; correlation. We have not aimed for a full discussion of the other criteria, and our findings should not be interpreted as proof of the role of these insects as vectors or key host carriers. Rather, we have discussed the existence of, and change in, a correlation between these insects and Buruli ulcer. Future work aims to explore spatial variation in the correlation between Buruli ulcer and the entire plant and animal communities, identifying any similarities between regions where the correlation exists, expanding on previous studies [37] which have focused on the community assemblage differences between *M. ulcerans* endemic and non-endemic regions of Ghana.

Despite these limitations, these results are consistent with previous research, which has shown that in Akonolinga the Nyong river is a risk factor for Buruli ulcer [30]. Our results agree with this conclusion; the main focus of suitable habitats for the insects in Akonolinga is the Nyong river, where the existence of large plants near the river banks provides appropriate habitat for Naucoridae and Belostomatidae to forage, develop and reproduce. Previous research has also implicated aquatic insects as important vectors in Akonolinga [14], including detection of *M. ulcerans* in the saliva of the insects.

In conclusion, we find a positive correlation between the abundance of Naucoridae and Belostomatidae suitable habitat and Buruli ulcer prevalence. This correlation is not constant, and changes in time and space. We interpret this as evidence consistent with that idea that Naucoridae and Belostomatidae may be locally important host carriers of *M. ulcerans* in certain conditions, their importance changing as the environmental conditions change, which would be expected in the situation of multi-host transmission.
